# Supplementary material for: Pulsed vector atomic magnetometer using an alternating fast-rotating field
Source: Nat Commun. 2025 Feb 5;16:1374. doi: 10.1038/s41467-025-56668-2 (PMC11799169; doi:10.1038/s41467-025-56668-2)
Supplement: Supplementary file 1 — Supplementary Information [file 41467_2025_56668_MOESM1_ESM.pdf]

# Supplementary Information: Pulsed Vector Atomic Magnetometer Using an Alternating Fast-Rotating Field

Tao Wang[1,2*], Wonjae Lee[1,3], Mark Limes[4,5], Tom Kornack[4], Elizabeth Foley[4], Michael Romalis[1*]

[1]Department of Physics, Princeton University, Princeton, 08544, NJ, USA.
[2]A*STAR Quantum Innovation Centre (Q.InC), Institute of Materials Research and Engineering (IMRE), Agency for Science, Technology and Research (A*STAR), 2 Fusionopolis Way, 08-03, Singapore, 138634, Republic of Singapore.
[3]Curent affiliation: Department of Physics, Harvard University, Cambridge, 02138 MA, USA.
[4]Twinleaf LLC, 300 Deer Creek Dr., Plainsboro, 08536, New Jersey, USA.
[5]Current affiliation: Virginia Tech, Blacksburg, Virginia 24061, USA.

*Corresponding author(s). E-mail(s): tao_wang@imre.a-star.edu.sg; romalis@princeton.edu;

## Supplementary Note 1. Hyperfine frequency phase shift

The Zeeman frequencies are split due to the nuclear magnetic moment. The hyperfine Landé g-factor can be written as [1]

$$\begin{aligned} g_F =& g_S \frac{F(F+1) - I(I+1) + S(S+1)}{2F(F+1)} \\ &- g_I \frac{F(F+1) + I(I+1) - S(S+1)}{2F(F+1)}. \end{aligned} \tag{S1}$$

If alkali atoms are exposed to the Earth's magnetic field (50 $\mu$T), the term involving the nuclear Landé g-factor $g_I$ cannot be disregarded. In the ground state with $F = 1$, the gyromagnetic ratio is $\gamma_{F=1} = -6.9778$ Hz/nT, while for $F = 2$, it is $\gamma_{F=2} = 7.0056$ Hz/nT. Consequently, atoms in these ground states precess in opposite directions, leading to a precession frequency difference of approximately $\omega_{hp} = 2\pi \times 1.39$ kHz in the Earth's field. Supplementary Figure 1 illustrates this frequency, which is measured when the atoms are not fully polarized and no rotating magnetic field is applied. This scenario can be effectively described using density matrix simulation.

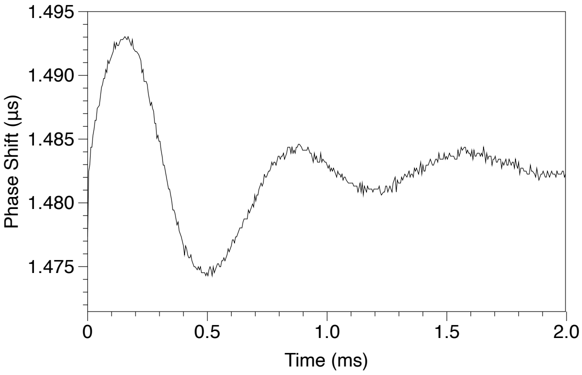


**Supplementary Fig. 1**: The pump beam power is reduced to reduce the spin polarization. The rotating field is turned off. The difference in the precession frequency for the F=1 and F=2 atoms induced an exponentially decaying sinusoidal phase shift at a frequency of $\omega_{hp} = 2\pi \times 1.39$ kHz.

# Supplementary Note 2. Derivation of spin dynamics based on rotating matrix

We assume the spins are fully polarized along the negative y-axis. The magnetic field initially points along the z-axis, while a rotating field originates from the x-axis and rotates counterclockwise in the xy-plane. We consider the behavior of polarization in a magnetic field, where it undergoes precession in response to the applied field. This magnetic field is composed of both a static and a rotating component. In the presence of these components, the polarization vector evolves according to specific rotations. The spin evolution, without considering spin relaxations, can be expressed as:

$$\mathbf{P}(t) = \mathcal{R}[\theta, \hat{z}] \cdot \mathcal{R}[\psi, \mathbf{B_{tot}}] \cdot (-\hat{y}), \tag{S2}$$

where $\mathcal{R}[\phi, \boldsymbol{v}]$ is a 3D rotation matrix for an anti-clockwise rotation of $\phi$ degrees around the vector $\boldsymbol{v} = \{i, j, k\}$,

$$\mathrm{R}[\phi, \boldsymbol{\nu}] = \begin{pmatrix} \frac{i^2+\left(j^2+k^2\right)\cos(\phi)}{i^2+j^2+k^2} & \frac{-k\sin(\phi)\sqrt{i^2+j^2+k^2}-ij\cos(\phi)+ij}{i^2+j^2+k^2} & \frac{j\sin(\phi)\sqrt{i^2+j^2+k^2}-ik\cos(\phi)+ik}{i^2+j^2+k^2} \\ \frac{k\sin(\phi)\sqrt{i^2+j^2+k^2}-ij\cos(\phi)+ij}{i^2+j^2+k^2} & \frac{\left(i^2+k^2\right)\cos(\phi)+j^2}{i^2+j^2+k^2} & \frac{-i\sin(\phi)\sqrt{i^2+j^2+k^2}-jk\cos(\phi)+jk}{i^2+j^2+k^2} \\ \frac{-j\sin(\phi)\sqrt{i^2+j^2+k^2}-ik\cos(\phi)+ik}{i^2+j^2+k^2} & \frac{i\sin(\phi)\sqrt{i^2+j^2+k^2}-jk\cos(\phi)+jk}{i^2+j^2+k^2} & \frac{\left(i^2+j^2\right)\cos(\phi)+k^2}{i^2+j^2+k^2} \end{pmatrix}$$

For the applied rotating field in the x-y plane, we have $B_x = B_m \sin(\omega_m t + \phi_x)$, $B_y = B_m \sin(\omega_m t + \phi_y)$, and $\mathrm{Abs}[\phi_x - \phi_y] = \pi/2$. Therefore, $\tilde{\mathbf{B}}_{\mathbf{tot}} = (B_m, 0, B_z - \omega_m/\gamma)$. Here, $\gamma$ represents the gyromagnetic ratio, and $\hat{x}$, $\hat{y}$ and $\hat{z}$ are the unit vectors along the x, y and z axes, respectively. We define $\theta = \omega_m t$ and $\psi = \gamma t\sqrt{B_m^2 + (B_z - \omega_m/\gamma)^2}$. Eventually, the spin projections can be written as:

$$\begin{aligned} P_x(t) =& \hat{x} \cdot \mathbf{P}(t) = \cos\omega_0 t \sin\omega_m t + \\ & \frac{\gamma B_z - \omega_m}{\omega_0} \sin\omega_0 t \cos\omega_m t, \end{aligned} \tag{S3}$$

$$P_z(t) = \hat{z} \cdot \mathbf{P}(t) = -\frac{\gamma B_m}{\omega_0} \sin\omega_0 t, \tag{S4}$$

where $\omega_0 = \gamma|\tilde{\mathbf{B}}_{\mathbf{tot}}|$. We have now included the first-order solution for the additional rotation caused by the transverse fields $b_x$ and $b_y$. This solution is derived based on the integration of the rotation angle, providing a more precise account of the transverse field effects in the measurement.

$$\begin{aligned} \psi \approx& \gamma t\sqrt{B_m^2 + b_x^2 + b_y^2 + (B_z - \omega_m/\gamma)^2} - \gamma t\frac{B_m b_y \cos\omega_m t}{\sqrt{B_m^2 + B_z^2}} \\ &+ \gamma t\frac{B_m b_x \sin\omega_m t}{\sqrt{B_m^2 + B_z^2}} \end{aligned} \tag{S5}$$

By inserting residual transverse magnetic fields $b_x$ and $b_y$ into Eq. S2, we can get

$$\begin{aligned} & P_x(t) \approx \cos\left(\omega_0 t + \gamma\frac{B_m b_y \cos\omega_m t - B_m b_x \sin\omega_m t}{\omega_m\sqrt{B_m^2 + B_z^2}}\right) \\ & \sin\omega_m t + \sin\left(\omega_0 t + \gamma\frac{B_m b_y \cos\omega_m t - B_m b_x \sin\omega_m t}{\omega_m\sqrt{B_m^2 + B_z^2}}\right) \\ & \cos\omega_m t \cdot \frac{\gamma B_z - \omega_m}{\omega_0}, \end{aligned} \tag{S6}$$

where $\omega_0 = \gamma|\tilde{\mathbf{B}}_{\mathbf{tot}}| = \gamma\sqrt{B_m^2 + b_x^2 + b_y^2 + (B_z - \omega_m/\gamma)^2}$.

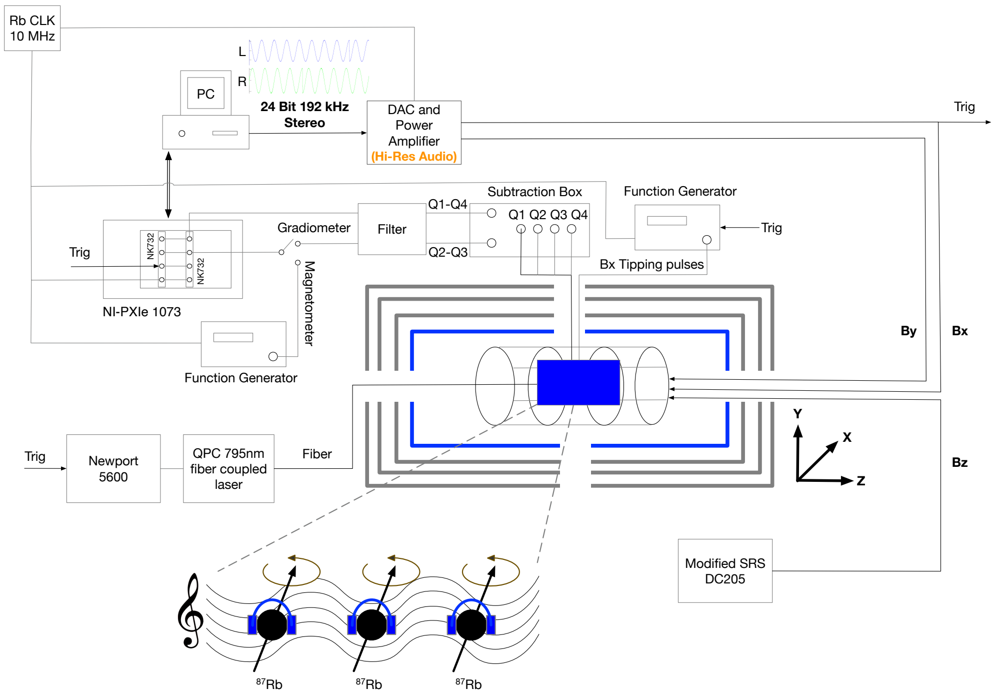


**Supplementary Fig. 2**: The experimental setup, which also demonstrates the method for measuring the magnetometer's sensitivity and gradiometer's sensitivity, is shown in this image.

# Supplementary Note 3. Setup

The setup is illustrated in the diagram shown in Supplementary Figure 2. The peak-altering fast rotating field was implemented using Python code to generate a 24-bit, 192 kHz stereo WAV file (Waveform Audio File Format), with the left and right channels corresponding to $B_x$ and $B_y$, respectively. The digital signal is then sent through a USB audio bridge to a balanced MQA DAC and power amplifier. Each channel is connected to a capacitor to filter out the DC signal before connecting to the coils.

# Supplementary Note 4. Noise Estimation

The optical polarimeter measures the spin projection along the transverse direction. The optical rotation can be written as [2]

$$\Theta = kP_x = \frac{1}{2} l r_e c f n P_x D(\nu), \tag{S7}$$

where $k = l r_e c f n D(\nu)/2$, $l$ is the length of probe beam travel through the cell, $r_e$ is the classical electron radius, $f$ is the typical oscillator strength of the D line transition,

$D(\nu) = (\nu - \nu_0)/[(\nu - \nu_0)^2 + (\Delta\nu/2)^2]$, where $\Delta\nu$ is the optical FWHM (full width at half maximum), $\nu_0$ is the frequency of the D line transition.

For a sinusoidal signal plus white Gaussian noise, the Cramer-Rao Low Bound (CRLB) of $\phi$ estimate can be written as [3]

$$var(\phi) \geq \frac{\rho^2}{A^2 t} \tag{S8}$$

Where $\rho$ is the power spectral density of the additive white Gaussian noise (AWGN) noise, $A$ is the amplitude of the sinusoidal signal, $t$ is the measurement time.

For an exponentially damped sinusoidal signal

$$\begin{aligned} x[n] &= A\sin(\omega_0 n + \phi) \cdot e^{-\frac{n}{T_2}} + w[n], \\ n &= 0, 1, 2...N-1. \end{aligned} \tag{S9}$$

where $T_2$ is the transverse relaxation time.

When $\omega_0 \gg 1/T_2$, we can consider the noise as quasi-AWGN for one period $2\pi/\omega_0$, the standard deviation of phase noise in one period can be written as

$$\sigma^2(\phi) = \frac{\rho^2}{A^2\Delta t} e^{\frac{2m\Delta t}{T_2}}, \ m = 0, 1, 2...M-1 \tag{S10}$$

where $\Delta t = 2\pi/\omega_0$. And $M$ is the number of the period $\Delta t$, the measurement time $t = M\Delta t$.

If the noise is not AWGN, we can model the data observation as

$$\mathbf{x} = \mathbf{H} \cdot \theta + \mathbf{w}, \tag{S11}$$

where $\mathbf{x}$ is the observation vector, $\mathbf{H}$ is the observation matrix, $\theta$ is the vector of parameters to be estimated, $\mathbf{w}$ is the noise vector, here it is a colored noise, the standard deviation of the noise increase exponentially

$$\mathbf{C} = \frac{\rho^2}{A^2\Delta t} \begin{bmatrix} 1 & 0 & 0 & \cdots & 0 \\ 0 & e^{\frac{2\Delta t}{T_2}} & 0 & \cdots & 0 \\ 0 & 0 & e^{\frac{4\Delta t}{T_2}} & & 0 \\ \vdots & \vdots & \vdots & \ddots & \vdots \\ 0 & 0 & 0 & \cdots & e^{\frac{2(M-1)\Delta t}{T_2}} \end{bmatrix} \tag{S12}$$

For a linear fitting $Y = a + b \cdot X$, the observation matrix $\mathbf{H}$ can be written as

$$\mathbf{H} = \begin{bmatrix} 1 & 0 \\ 1 & 1 \\ 1 & 2 \\ \vdots & \vdots \\ 1 & M-1 \end{bmatrix} \tag{S13}$$

$\theta_{\mathbf{1}} = [a, b]^T$. The covariance of the Minimum-Variance Unbiased (MVU) estimator can be written as [3]

$$\begin{aligned} \mathbf{C}_{\theta_1} &= (\mathbf{H}^T \mathbf{C}^{-1} \mathbf{H})^{-1} \\ &= \frac{\rho^2}{A^2 \Delta t} \frac{1}{M_1 M_2 - M_0^2} \begin{bmatrix} M_1 & -M_0 \\ -M_0 & M_2 \end{bmatrix} \end{aligned} \tag{S14}$$

where

$$\begin{aligned} M_0 &= \sum_{m=0}^{M-1} m e^{-\frac{2m\Delta t}{T_2}} \\ &= \frac{z^2 - M z^{2M} - z^{2+2M} + M z^{2+2M}}{(z^2-1)^2} \end{aligned} \tag{S15}$$

$$\begin{aligned} M_1 &= \sum_{m=0}^{M-1} m^2 e^{-\frac{2m\Delta t}{T_2}} \\ &= \frac{-M^2 z^{2M}}{1-z^2} + \frac{z^2(1 - z^{2M} - 2M z^{2M})}{(1-z^2)^2} \\ &+ \frac{2z^4(1 - z^(2M))}{(1-z^2)^3} \end{aligned} \tag{S16}$$

$$M_2 = \sum_{m=0}^{M-1} e^{-\frac{2m\Delta t}{T_2}} = \frac{1 - z^{2M}}{1 - z^2} \tag{S17}$$

where $z = e^{-\Delta t/T_2}$, the slope is defined as the change of the phase shift in the unit of time in unit measurement time $t$, so the standard deviation of phase needs to be divided by $2\pi$, the variance of the slope $b$ can be written as

$$\sigma^2(b) \geq \frac{\rho^2}{(2\pi)^2 A^2 \Delta t} \frac{M_2}{M_1 M_2 - M_0^2}. \tag{S18}$$

We define

$$\begin{aligned} \kappa_1(M) = \frac{M_2}{M_1 M_2 - M_0^2} = \\ \frac{(1-z^2)^3(1-z^{2M})}{z^2 + z^{2+4M} - z^{2M}(2z^2 + M^2(1-z^2)^2)} \end{aligned} \tag{S19}$$

The measured slope of the phase shift $b = \Delta\omega/\omega_0$, and the total magnetic field equals $(\Delta\omega + \omega_0)/\gamma$. The power spectral density of the noise of the $\Delta\omega$ measurement can be written as

$$\rho(\Delta\omega) \geq \frac{\rho}{A\Delta t}\sqrt{2M \cdot \kappa_1(M)}. \tag{S20}$$

We can get when $t = M\Delta t \approx 2T_2$, the power spectral density $(\rho(\Delta\omega))$ reaches its minimum. So the optimized measurement time is approximately $2T_2$.

When there is no relaxation, $T_2 \to \infty$, $\kappa_1(M) \to 12/M^3$, so the standard deviation of the frequency can be written as

$$\sigma^2(\Delta\omega) = \omega_0^2 \sigma^2(b) \geq \frac{12\rho^2}{A^2 t^3} \tag{S21}$$

If the measurement time is long enough $M \to \infty$, $\kappa_1 \to (2\Delta t/T_2)^3$

$$\sigma^2(\Delta\omega) \geq \frac{8\rho^2}{A^2 T_2^3} \tag{S22}$$

Now, we consider the standard deviation of the amplitude of the sinusoidal signal

$$\begin{aligned} Y[m] &= A\cos(\omega_m m + \phi_0) + \mathbf{w} \\ &= A\cos\phi_0 \cos(\omega_m m) - A\sin\phi_0 \sin(\omega_m m) + \mathbf{w} \\ &= a_1 \cos(\omega_m m) + -a_2 \sin(\omega_m m) + \mathbf{w} \\ &,\ m = 0, 1, 2 \ldots M-1. \end{aligned} \tag{S23}$$

We can have $A = \sqrt{a_1^2 + a_2^2}$, and

$$\begin{aligned} \sigma(A) &= \sqrt{\left[\frac{\partial A}{\partial a_1}\sigma(a_1)\right]^2 + \left[\frac{\partial A}{\partial a_2}\sigma(a_2)\right]^2} \\ &= \sqrt{[\cos\phi_0 \cdot \sigma(a_1)]^2 + [\sin\phi_0 \cdot \sigma(a_2)]^2} \end{aligned} \tag{S24}$$

If we choose $\phi_0 = 45°$, $\sigma(A) = \sqrt{([\sigma(a_1)]^2 + [\sigma(a_2)]^2)/2}$.

The observation matrix $\mathbf{H}$ can be written as

$$\mathbf{H} = \begin{bmatrix} 1 & 0 \\ \cos[\omega_m] & -\sin[\omega_m] \\ \cos[2\omega_m] & -\sin[2\omega_m] \\ \vdots & \vdots \\ \cos[(M-1)\omega_m] & -\sin[(M-1)\omega_m] \end{bmatrix} \tag{S25}$$

and $\boldsymbol{\theta_2} = [a_1, a_2]^T$. The covariance of the Minimum-Variance Unbiased (MVU) estimator can be written as [3]

$$\begin{aligned} \mathbf{C}_{\boldsymbol{\theta_2}} &= (\mathbf{H}^T \mathbf{C}^{-1} \mathbf{H})^{-1} \\ &= \frac{\rho^2}{A^2 \Delta t} \frac{1}{Q_1 Q_2 - Q_0^2} \begin{bmatrix} Q_1 & Q_0 \\ Q_0 & Q_2 \end{bmatrix}, \end{aligned} \tag{S26}$$

where

$$\begin{aligned} Q_0 &= \sum_{m=0}^{M-1} \frac{1}{2} e^{-\frac{2m\Delta t}{T_2}} \sin(2\omega_m m) \\ &\approx 0 \end{aligned} \tag{S27}$$

$$
\begin{aligned}
Q_1 &= \sum_{m=0}^{M-1} \frac{1}{2} e^{-\frac{2m\Delta t}{T_2}} [1 - \cos(2\omega_m m)] \\
&\approx \frac{1 - e^{-\frac{2M\Delta t}{T_2}}}{2(1 - e^{-\frac{2\Delta t}{T_2}})}
\end{aligned} \tag{S28}
$$

$$
\begin{aligned}
Q_2 &= \sum_{m=0}^{M-1} \frac{1}{2} e^{-\frac{2m\Delta t}{T_2}} [1 + \cos(2\omega_m m)] \\
&\approx \frac{1 - e^{-\frac{2M\Delta t}{T_2}}}{2(1 - e^{-\frac{2\Delta t}{T_2}})}
\end{aligned} \tag{S29}
$$

We define

$$
\kappa_2(M) = \frac{\sqrt{(Q_1^2 + Q_2^2)/2}}{Q_1 Q_2 - Q_0^2} = \frac{2(1 - z^2)}{1 - z^{2M}} \tag{S30}
$$

The power spectral density of the noise of the amplitude measurement, and the transverse magnetic fields are proportional to the measured amplitude of phase shift. The power spectral density of the amplitude measurement noise can be written as

$$
\rho(A) \geq \frac{\rho}{A} \sqrt{2M \cdot \kappa_2(M)}. \tag{S31}
$$

Different from the total magnetic field, the power spectral density noise $\rho(A)$ of the transverse magnetic field measurement keeps increasing with a longer measurement time.

When there is no relaxation ($T_2 \to \infty$), $Q_1, Q_2 \to M/2$, $\kappa_2(M) \to 2/M$. So the standard deviation of the amplitude estimate can be written as

$$
\sigma^2(A) \geq \frac{2\rho^2}{A^2 t} \tag{S32}
$$

If measurement time is long enough $M \to \infty$, $Q_1, Q_2 \to T_2/(4\Delta t)$, $\kappa_2 \to 4\Delta t/T_2$

$$
\sigma^2(A) \geq \frac{\rho^2}{A^2 \Delta t} \frac{Q_1}{Q_1 Q_2 - Q_0^2} = \frac{4\rho^2}{A^2 T_2} \tag{S33}
$$

# Supplementary Note 5. Fundamental sensitivity of FRF vector magnetometers

The noise of the measurement of the precession signal is from the photon shot noise of the probe beam, and the power spectral density of the shot noise in the rotation angle can be written as [4]

$$
\rho_\Theta = \sqrt{\frac{1}{2\Phi_{pr}}}, \tag{S34}
$$

where $\Phi_{pr}$ is the total flux of photons of the probe beam per unit of time.

The limit of the phase estimation of the sinusoidal signal $k\sin(\omega t+\psi)+w$ can be given by Cramer Rao Lower Bound (CRLB) [3]

$$\sigma_\psi \geq \frac{\rho_\Theta}{k\sqrt{t}}, x \tag{S35}$$

where $w$ AWGN, whose power spectral density is $\rho_\Theta$. In the experiment, the free spin precession signal damps exponentially with a transverse relaxation time $T_2$; if precession frequency $\omega_0 \gg 1/T_2$, the phase shift noise can be considered as white Gaussian noise during each precessing period $\Delta t = 2\pi/\omega_0$.

$$\sigma_\psi \geq \frac{\rho_\Theta}{k\sqrt{\Delta t}} e^{\frac{m\Delta t}{T_2}},\ m = 0, 1, 2...M-1, \tag{S36}$$

where $M$ is the period number of $\Delta t$, and the measurement time $t = M\Delta t$. After the phase shift is measured, the slope of the phase shift is fitted to obtain the total magnetic field. And the slope equals the $(\omega-\omega_0)/\omega_0$, the power spectral density of the noise of $\omega$ measurement can be calculated by the Minimum-Variance Unbiased (MVU) estimation (Sec. Supplementary Note 4. of the supplemental material.)

$$\rho(\omega) \geq \frac{\rho_\Theta}{k\Delta t}\sqrt{2M\cdot\kappa_1(M)}, \tag{S37}$$

where

$$\kappa_1(M) = \frac{(1-z^2)^3(1-z^{2M})}{z^2+z^{2+4M}-z^{2M}(2z^2+M^2(1-z^2)^2)}, \tag{S38}$$

where $z = e^{-\Delta t/T_2}$. The power spectral density $\rho(\omega)$ in Eq. S37 has a minimum when the measurement time $t \approx 2T_2$. And the total magnetic field measurement sensitivity is given by

$$\delta B_{tot} \geq \frac{4}{\gamma k T_2\sqrt{\Phi_{pr}}}. \tag{S39}$$

For the transverse magnetic fields, $b_x$ and $b_y$ are measured by fitting the amplitude of $\sin\omega_m t$ and $\cos\omega_m t$ in the phase shift, respectively.

The power spectral density of the noise of the amplitude estimation of a sinusoidal signal can be written as (Sec. Supplementary Note 4. of the supplemental material.)

$$\rho(A) \geq \frac{\rho_\Theta}{k}\sqrt{2M\cdot\kappa_2(M)}. \tag{S40}$$

Where $\kappa_2$ is given by

$$\kappa_2(M) = \frac{2(1-z^2)}{1-z^{2M}} \approx \frac{4\Delta t}{T_2(1-e^{-\frac{2t}{T_2}})}, \tag{S41}$$

and $\delta B_{tran} = \rho(A) \cdot \omega_m \sqrt{B_z^2 + B_m^2}/(\gamma B_m)$, we can have the transverse magnetic field measurement sensitivity

$$\delta B_{tran} \geq \frac{\omega_m \csc\theta}{\gamma k \sqrt{\Phi_{pr}}} \sqrt{M \cdot \kappa_2(M)}. \tag{S42}$$

Differing from the total magnetic field measurement, the power spectral density of the transverse magnetic field measurement noise keeps increasing with a longer measurement time. If the measurement time is short enough, $\kappa_2 \to 2\Delta t/t$, and the transverse magnetic field measurement sensitivity is given by

$$\delta B_{tran} \geq \frac{\sqrt{2}\omega_m \csc\theta}{\gamma k \sqrt{\Phi_{pr}}}. \tag{S43}$$

## Supplementary Note 6. Fundamental sensitivity of the sequential modulation vector magnetometers

As mentioned in Sec. Supplementary Note 7., the sequential modulation vector magnetometers measure the transverse magnetic field by altering $B_m$ polarity along the x-axis and compare the difference of the Larmor precession frequencies.

$$\begin{aligned}&\omega_1 - \omega_2 = \\ &\gamma \left[ \sqrt{B_z^2 + (B_m + b_x)^2} - \sqrt{B_z^2 + (-B_m + b_x)^2} \right]\end{aligned} \tag{S44}$$

And the uncertainty of Larmor frequency can be written as

$$\begin{aligned}\sqrt{2}\delta\omega &= \frac{1}{2} \frac{2b_x + 2B_m}{\sqrt{B_z^2 + (B_m + b_x)^2}} \gamma \delta b_x \\ &- \frac{1}{2} \frac{2b_x - 2B_m}{\sqrt{B_z^2 + (B_m - b_x)^2}} \gamma \delta b_x \\ &\approx \frac{2\gamma B_m}{\sqrt{B_z^2 + B_m^2}} \delta b_x = 2\gamma \sin\theta \cdot \delta b_x.\end{aligned} \tag{S45}$$

Moreover, sequential modulation vector magnetometers need four times longer time to measure all three vector components, therefore, the fundamental sensitivity of the transverse magnetic field can be written as

$$\delta B_{tran\_SM} = \rho(b_x) = \frac{\delta\Delta\omega}{\sin\theta} \sqrt{2t} = \frac{4\sqrt{2}}{\gamma k T_2 \sin\theta \sqrt{\Phi_{pr}}} \tag{S46}$$

If we assume $\omega_m = \pi/T_2$, The sensitivity of the transverse magnetic field of our fast rotating field vector magnetometer can be written as

$$\delta B_{tran\_FRF} = \frac{2\sqrt{2}\pi}{\gamma k T_2 \sin\theta \sqrt{\Phi_{pr}}} \tag{S47}$$

Suppose we further reduce the frequency of the rotating field. In that case, the fast-rotating field vector magnetometer has a comparable or even better sensitivity to measuring the transverse magnetic fields than the sequential modulation vector magnetometers.

## Supplementary Note 7. Heading error in the sequential modulation vector magnetometers

Fig. 3 illustrates the setup of sequential modulation vector magnetometers, where a very slow modulation is applied to maintain quasi-static conditions. To measure the residual magnetic field ($b_x$) along the x-direction, a constant magnetic field ($B_m$) is applied along the positive x-axis, and the precession frequency ($\omega_1$) is recorded. Then, a constant $B_m$ is applied along the negative x-axis, and the precession frequency ($\omega_2$) is recorded. The value of $b_x$ is determined by comparing $\omega_1$ and $\omega_2$. Similarly, the residual magnetic field along the y-axis ($b_y$) can be measured.

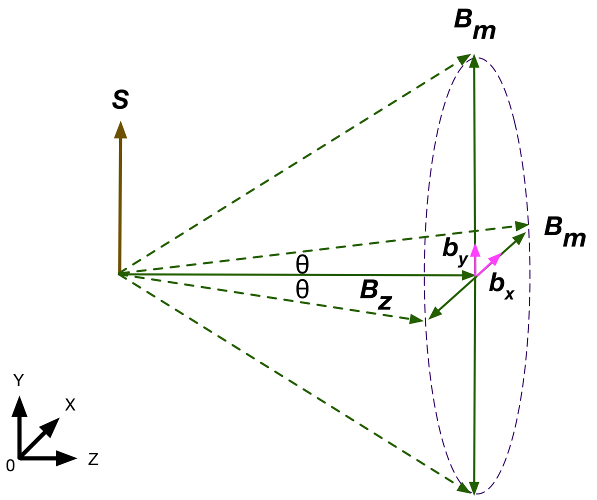


**Supplementary Fig. 3**: Sequential modulation of the vector magnetometers.

We assume that the spin evolution satisfies the quasi-static condition for each measurement. According to the analytical solution for the static heading error [5], the

fictitious magnetic field caused by the static heading error can be expressed as [5]

$$B_{SH} = \mathcal{B}_{\mathcal{H}} \sin\beta, \tag{S48}$$

where $\mathcal{B}_{\mathcal{H}}$ is the maximum static heading error,

$$\mathcal{B}_{\mathcal{H}} \approx \frac{P(7+P^2)}{5+3P^2}\frac{3h\gamma B_{tot}^2}{4\pi A_{hf}}, \tag{S49}$$

where $P$ is spin polarization, $A_{hf} = h\cdot 3.417$ GHz is the hyperfine structure constant for ground state [6], $h$ is Planck constant, $\beta$ is the angle of the pump beam relative to the field. The spin projection along the total magnetic field determines the static heading error.

If the magnetometer operates in the sequential modulation vector magnetometer mode[7], with atoms polarized along the y-axis, it experiences a heading error of $\mathcal{B}_H \sin\theta$ when $B_m$ is along the y-plus axis, and a heading error of $-\mathcal{B}_H \sin\theta$ when $B_m$ is along the y-minus axis. These heading errors along the y-axis can impact the measurement of $b_y$. Similarly, if the atoms are polarized along the x-axis, there will be a systematic heading error effect along the x-axis.

If the magnetometer works with a very slow rotating field ($\omega_m \ll 1/T_2$), and the atoms are continuously pumped along the negative y-axis. We can consider the spin evolution to satisfy the quasi-static condition for each measurement, as shown in Fig. 4. When spins are polarized along the y-axis initially for each measurement, and the total magnetic field is $\mathbf{B_{tot}}$, the spin has a projection $\mathbf{S}_\parallel$ that is parallel to the total magnetic field, and a spin projection $\mathbf{S}_\perp$ that is perpendicular to the total magnetic field.

The spin projection $\mathbf{S}_\parallel$ along the total magnetic field varies as the total magnetic field rotates at the frequency of $\omega_m$, $\mathbf{S}_\parallel = \mathbf{S}\sin\theta\cos\omega_m t$. If there are residual magnetic fields $b_x$ and $b_y$ in the x and y direction, respectively, and the rotating magnetic field is applied in the x-y plane, the precession frequency of the total magnetic field can be written as

$$\begin{aligned}
&\gamma[B_z^2 + (b_x + B_m\cos\omega_m t)^2 + (b_y + B_m \sin\omega_m t)^2]^{1/2} \\
&+ \frac{\gamma B_m \mathcal{B}_{\mathcal{H}}}{\sqrt{B_m^2+B_z^2}}\cos\omega_m t \\
&\approx \gamma\sqrt{B_m^2+B_z^2} + \frac{\gamma B_m b_x}{\sqrt{B_m^2+B_z^2}}\cos\omega_m t \\
&+ \frac{\gamma B_m b_y}{\sqrt{B_m^2+B_z^2}}\sin\omega_m t + \frac{\gamma B_m \mathcal{B}_{\mathcal{H}}}{\sqrt{B_m^2+B_z^2}}\cos\omega_m t.
\end{aligned} \tag{S50}$$

The heading error effect is equivalent to a residual magnetic field along the x-direction $b_x$, and it equals $\mathcal{B}_H$, which doesn't depend on $\theta$. For instance, a $^{87}$Rb cell in a total magnetic field equals 50 $\mu$T and $P = 1$, the dynamic heading error is expected to cause a fictitious transverse magnetic field of 7.7 nT in the transverse direction. [1]

[1] Here, we assume that the atoms are polarized along the y-direction, and the rotating magnetic field begins in the y-direction. If the rotating magnetic field were to start from the x-direction, it would result in a fictitious magnetic field equivalent to $b_y$.

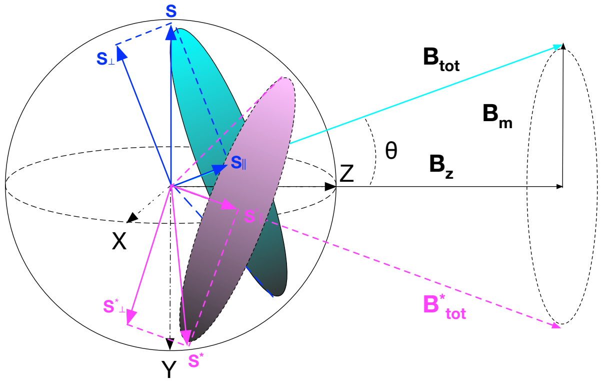


**Supplementary Fig. 4**: In the sequential modulation vector magnetometers, a slow rotating field induces heading errors in transverse directions based on the quasi-static evolution of the atomic spins.

# Supplementary Note 8. Density Matrix Simulation

In an alkali vapor cell, a density matrix is more suitable for describing an ensemble of spins in a mixed state compared to a single wave function. For a system with $N$ atoms, the density matrix operator can be expressed as:

$$\rho = \frac{1}{N}\sum_i |\psi_i\rangle\langle\psi_i|, \tag{S51}$$

where $|\psi_i\rangle$ is a single wave function. The ground state density matrix evolves as [8]

$$\begin{aligned}\frac{\mathrm{d}}{\mathrm{d}t}\rho =& \frac{1}{i\hbar}[H,\rho] + R_{OP}[\varphi(1+2\mathbf{s}\cdot\mathbf{S})-\rho] \\ &+ R_{SE}[\varphi(1+4\langle\mathbf{S}\rangle\cdot\mathbf{S})-\rho] \\ &+ R_{SD}[\varphi-\rho],\end{aligned} \tag{S52}$$

where

$$\varphi = \frac{1}{4}\rho + \mathbf{S}\cdot\rho\mathbf{S}, \tag{S53}$$

$R_{OP}$ is the optical pumping rate, $R_{SE}$ is the spin-exchange rate, $\langle\mathbf{S}\rangle$ is the expect value of spin, $R_{SD}$ is the spin-destruction rate. and the optical pumping-related term is an approximation based on the fact that there is high-pressure buffer gas in the cell. The Hamiltonian describing the interaction with the magnetic field is [9]

$$H = A_{hf}\mathbf{I}\cdot\mathbf{S} + g_S\mu_B\mathbf{S}\cdot\mathbf{B} - \frac{\mu_I}{I}\mathbf{I}\cdot\mathbf{B}. \tag{S54}$$

Where $\mathbf{I}$ is nuclear spin, $\mathbf{S}$ is electron spin, The measured spin projection can be calculated by density matrix as a weighted sum of coherences oscillating at different

Zeeman frequencies

$$\langle \mathbf{S} \rangle = Tr[\rho \mathbf{S}] = \sum_{Fm'=m\pm1} A_{Fm,m'} \rho_{Fm,m'}, \tag{S55}$$

where $\rho_{Fm,m'}$ represents the off-diagonal coherence element of the density matrix in the coupled basis $|Fm\rangle$, with $A_{Fm,m'}$ as its amplitude. For $F = 2$, there are four coherence terms corresponding to $\Delta m = \pm 1$. We can derive the time-domain signal and then identify the roots to obtain the zero-crossings of the precession signal. The phase shifts between these zero-crossings and those of the reference signal $(N\frac{2\pi}{\omega_0})$ are then calculated. The fitting model further analyzes these phase shifts as a function of measurement time to obtain the fitted values for offset, slope, and the amplitudes of $\sin \omega_m$, $\cos \omega_m$, $\sin 2\omega_m$, $\cos 2\omega_m$, $\sin \omega_{hp}$, and $\cos \omega_{hp}$.

$$\begin{aligned} \delta\Phi(t) =& \Phi_{off} + \Phi_{tot} \cdot t + \Phi_x \sin \omega_m t + \Phi_y \cos \omega_m t \\ &+ \Phi_{2s} \sin 2\omega_m t + \Phi_{2c} \cos 2\omega_m t \\ &+ \Phi_{hps} \sin \omega_{hp} t + \Phi_{hpc} \cos \omega_{hp} t \end{aligned} \tag{S56}$$

The simulation results based on the density matrix and the experimental results are shown in Sec. Supplementary Note 8..

# Supplementary Tables

Some progress in achieving high dynamic range vector magnetometers is listed in Supplementary Table 1.

To investigate the heading error, the sensor is rotated 90° counterclockwise about the z-axis, as shown in Supplementary Figure 5. A horizontal rotation stage in the xy-plane allows adjustment of the relative angle between the pump beams. Note that in the following tables, the x and y labels for the heading error are swapped. In the main paper, we convert these heading error results back to the x and y axes to maintain consistency.

**Supplementary Table 1**: Vector atomic magnetometers and their performances.

| Year | Type | Tested field ($\mu$T) | Total field sensitivity (fT/$\sqrt{\text{Hz}}$) | Fractional resolution (1/$\sqrt{\text{Hz}}$) | Polar angle sensitivity (nrad/$\sqrt{\text{Hz}}$) | | Ref |
|---|---|---|---|---|---|---|---|
| 2004 | Unshielded SERF magnetometer | 50 | 1,000 (down to 10 Hz) | $2\times10^{-8}$ | — | — | [10] |
| 2006 | Variometer based on scalar cesium magnetometer | 50 | 4,000 (estimated) | $8\times10^{-8}$ | $2\times10^{2}$ (estimated) | $2\times10^{2}$ (estimated) | [11] |
| 2014 | All optical vector NMOR atomic magnetometer | 1.5 | 65 (down to 0.5 Hz) | $4\times10^{-8}$ | $5\times10^{5}$ | only measure one polar angle | [12] |
| 2015 | Multi-laser beam atomic vector magnetometer based on free spin precession | 1 | 143 (estimated) | $1\times10^{-7}$ | $4\times10^{4}$ (estimated) | $4\times10^{4}$ (estimated) | [13] |
| 2019 | Spin self-sustaining Larmor precession vector magnetometer | 0.2 | 780 (down to 2 Hz) | $3.9\times10^{-6}$ | $3.5\times10^{3}$ | $3.5\times10^{3}$ | [14] |
| 2019 | Voigt-effect-based vector magnetometer | 0.26 | 400 (down to 2 Hz) | $2\times10^{-6}$ | $8\times10^{3}$ (down to 10 Hz) | $8\times10^{3}$ (down to 10 Hz) | [15] |
| 2023 | Machine learning assisted NMOR vector magnetometer | 0.14 | 100 (down to 20 Hz) | $7\times10^{-7}$ | $10^{5}$ (down to 16 Hz) | $2\times10^{5}$ (down to 18 Hz) | [16] |
| - | FRF vector magnetometer with CARFM modulation | 35 | 35 (down to 1 Hz) | $1\times10^{-9}$ | 6 (down to 0.1 Hz) | 6 (down to 0.1 Hz) | This work |

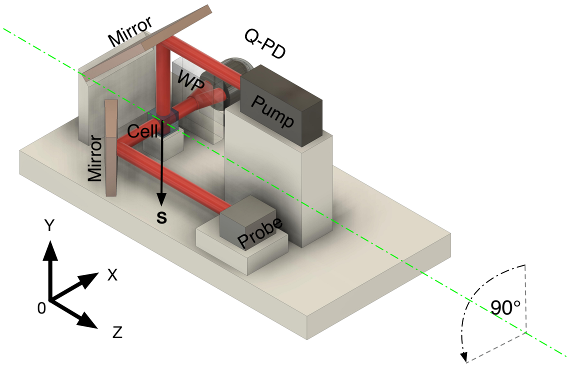


**Supplementary Fig. 5**: Rotate the sensor head by 90°, allowing the pump beam to rotate on a stage in the xz-plane. The xyz axes are defined by the coils.

**Supplementary Table 2**: The composition of the slope of the fitting result, and their sign dependence on the phases of the rotating field.

| **Shot No.** | $\phi_x$ | $\phi_y$ | Slope (nT) $\propto \Phi_{tot}$ |
|---|---|---|---|
| 1 | 90° | 0° | $\delta b_z, B_{SH}, B_{Berry}(+), B_{PD}(+)$ |
| 2 | 90° | 180° | $\delta b_z, B_{SH}, B_{Berry}(-), B_{PD}(+)$ |
| 3 | 270° | 180° | $\delta b_z, B_{SH}, B_{Berry}(+), B_{PD}(-)$ |
| 4 | 270° | 360° | $\delta b_z, B_{SH}, B_{Berry}(-), B_{PD}(-)$ |

**Supplementary Table 3**: Analytical model of dynamic heading error for $\mathbf{B_m}$ initiated from the Y-Axis and X-Axis

| | $\mathbf{B_m}$ initiates from Y-axis | $\mathbf{B_m}$ initiates from X-axis |
|---|---|---|
| **Shot No.** | $B_{DH}$ | $B_{DH}$ |
| 1 | $\mathcal{B}_{\mathcal{H}} \sin(\beta + \theta)$ | $\mathcal{B}_{\mathcal{H}} \cos\theta \sin\beta$ |
| 2 | $\mathcal{B}_{\mathcal{H}} \sin(\beta + \theta)$ | $\mathcal{B}_{\mathcal{H}} \cos\theta \sin\beta$ |
| 3 | $\mathcal{B}_{\mathcal{H}} \sin(\beta - \theta)$ | $\mathcal{B}_{\mathcal{H}} \cos\theta \sin\beta$ |
| 4 | $\mathcal{B}_{\mathcal{H}} \sin(\beta - \theta)$ | $\mathcal{B}_{\mathcal{H}} \cos\theta \sin\beta$ |
| **Average** | $\mathcal{B}_{\mathcal{H}} \cos\theta \sin\beta$ | $\mathcal{B}_{\mathcal{H}} \cos\theta \sin\beta$ |

Supplementary Table 2 shows that the slope of the fitting result reflects contributions from four effects: a small magnetic field deviation $\delta b_z$ between the reference frequency and $B_z$ (where $\delta b_z = B_z - \omega_0/\gamma$), Berry's phase shift ($B_{Berry}$), and dynamic heading error ($B_{DH} = B_{SH} + B_{PD}$).

To distinguish these effects:

⋆ $\delta b_z$ and $B_{SH}$ can be calculated by averaging four shots, with further distinction achieved by reversing the pumping direction, as the heading error sign depends on the spin polarization direction, whereas the residual magnetic field does not.

⋆ $B_{PD}$ is calculated by averaging the four shots with coefficients +1, +1, -1, and -1.

⋆ $B_{Berry}$ can be determined by averaging the four-shot results, applying coefficients of +1, -1, +1, and -1.

Supplementary Table 3 presents the analytical model for dynamic heading error, based on our finding that this error depends on the initial angle between the spin polarization and $\mathbf{B_{tot}}$, which remains constant. We define $\Theta$ as the angle by which $\mathbf{B_{tot}}$ deviates from the position where $\mathbf{S}$ is perpendicular to $\mathbf{B_{tot}}$, with $B_{DH} = \mathcal{B}_{\mathcal{H}} \sin\Theta$. The definitions of $\beta$ and $\theta$ are provided in the main paper.

Supplementary Tables 4 and 5 present measurements taken without switching the rotating field during each panorama measurement, effectively eliminating eddy currents. In each panorama, the initial 8 shots (corresponding to the measured eddy current time constant of 10.4 ms) were discarded to ensure the eddy current was fully damped.

Supplementary Tables 6 and 7 show measurements using the four-shot scheme proposed in this study. While the "peak-altering" largely cancels the eddy current, minor imperfections in the switch can introduce residual eddy effects within each shot, which are subsequently canceled by averaging across the four shots.

**Supplementary Table 4**: **Total magnetic field** measurement without altering the phases of the rotating field during each panorama measurement. All the slope values are subtracted by 0.5 nT to remove $\delta b_z$. The dynamic heading error highly depends on the initial alignment between the rotating field and spin polarization. Suppose the rotating field initially starts perpendicularly to the spin polarization because there is no spin polarization along the total magnetic field. In that case, there is no heading error contribution from the rotating field. The average of four slopes results in a static heading error.

| $\mathbf{B_m}$ initiates from Y-axis | | **Experiment (in units of nT)** | | | | | **Density Matrix (in units of nT)** | | | | |
|---|---|---|---|---|---|---|---|---|---|---|---|
| **Shot No.** | $\beta$ | **Slope** | $B_{Berry}$ | $B_{DH}$ | $B_{SH}$ | $B_{PD}$ | **Slope** | $B_{Berry}$ | $B_{DH}$ | $B_{SH}$ | $B_{PD}$ |
| 1 | 24° | 9.7 | 5.2 | 4.6 | 2.2 | 2.4 | 10.5 | 4.8 | 5.8 | 3.1 | 2.7 |
| 2 | | -0.7 | -5.2 | 4.5 | 2.2 | 2.3 | 1.0 | -4.8 | 5.8 | 3.1 | 2.7 |
| 3 | | 4.9 | 5.2 | -0.2 | 2.2 | -2.4 | 5.1 | 4.8 | 0.4 | 3.1 | -2.7 |
| 4 | | -5.3 | -5.2 | -0.2 | 2.2 | -2.3 | -4.4 | -4.8 | 0.4 | 3.1 | -2.7 |
| **Average** | | **2.2** | **0.0** | **2.2** | **2.2** | **0.0** | **3.1** | **0.0** | **3.1** | **3.1** | **0.0** |
| 1 | 24° Reverse pump | 0.0 | 5.1 | -5.1 | -2.6 | -2.5 | -1.0 | 4.8 | -5.8 | -3.1 | -2.7 |
| 2 | | -10.1 | -5.1 | -5.0 | -2.6 | -2.5 | -10.5 | -4.8 | -5.8 | -3.1 | -2.7 |
| 3 | | 5.0 | 5.1 | -0.1 | -2.6 | 2.5 | 4.4 | 4.8 | -0.4 | -3.1 | 2.7 |
| 4 | | -5.2 | -5.1 | -0.1 | -2.6 | 2.5 | -5.1 | -4.8 | -0.4 | -3.1 | 2.7 |
| **Average** | | **-2.6** | **0.0** | **-2.6** | **-2.6** | **0.0** | **-3.1** | **0.0** | **-3.1** | **-3.1** | **0.0** |
| 1 | 0° | 7.4 | 5.1 | 2.3 | -0.3 | 2.6 | 7.7 | 4.8 | 3.0 | 0.0 | 3.0 |
| 2 | | -2.8 | -5.1 | 2.3 | -0.3 | 2.6 | -1.8 | -4.8 | 3.0 | 0.0 | 3.0 |
| 3 | | 2.2 | 5.1 | -2.9 | -0.3 | -2.6 | 1.8 | 4.8 | -3.0 | 0.0 | -3.0 |
| 4 | | -7.9 | -5.1 | -2.8 | -0.3 | -2.6 | -7.7 | -4.8 | -3.0 | 0.0 | -3.0 |
| **Average** | | **-0.3** | **0.0** | **-0.3** | **-0.3** | **0.0** | **0.0** | **0.0** | **0.0** | **0.0** | **0.0** |
| 1 | 0° Reverse pump | 2.9 | 5.0 | -2.1 | 0.4 | -2.5 | 1.8 | 4.8 | -3.0 | 0.0 | -3.0 |
| 2 | | -7.2 | -5.0 | -2.2 | 0.4 | -2.6 | -7.7 | -4.8 | -3.0 | 0.0 | -3.0 |
| 3 | | 7.9 | 5.0 | 2.9 | 0.4 | 2.5 | 7.7 | 4.8 | 3.0 | 0.0 | 3.0 |
| 4 | | -2.1 | -5.0 | 2.9 | 0.4 | 2.6 | -1.8 | -4.8 | 3.0 | 0.0 | 3.0 |
| **Average** | | **0.4** | **0.0** | **0.4** | **0.4** | **0.0** | **0.0** | **0.0** | **0.0** | **0.0** | **0.0** |
| 1 | -24° | 4.9 | 5.1 | -0.2 | -2.7 | 2.5 | 4.4 | 4.7 | -0.4 | -3.1 | 2.7 |
| 2 | | -5.5 | -5.1 | -0.4 | -2.7 | 2.3 | -5.1 | -4.7 | -0.4 | -3.1 | 2.7 |
| 3 | | 0.0 | 5.1 | -5.1 | -2.7 | -2.5 | -1.0 | 4.7 | -5.8 | -3.1 | -2.7 |
| 4 | | -10.0 | -5.1 | -4.9 | -2.7 | -2.3 | -10.5 | -4.7 | -5.8 | -3.1 | -2.7 |
| **Average** | | **-2.7** | **0.0** | **-2.7** | **-2.7** | **0.0** | **-3.1** | **0.0** | **-3.1** | **-3.1** | **0.0** |
| 1 | -24° Reverse pump | 6.3 | 5.3 | 1.1 | 3.2 | -2.1 | 5.1 | 4.7 | 0.4 | 3.1 | -2.7 |
| 2 | | -4.4 | -5.3 | 0.9 | 3.2 | -2.3 | -4.4 | -4.7 | 0.4 | 3.1 | -2.7 |
| 3 | | 10.5 | 5.3 | 5.3 | 3.2 | 2.1 | 10.5 | 4.7 | 5.7 | 3.1 | 2.7 |
| 4 | | 0.2 | -5.3 | 5.5 | 3.2 | 2.3 | 1.0 | -4.7 | 5.7 | 3.1 | 2.7 |
| **Average** | | **3.2** | **0.0** | **3.2** | **3.2** | **0.0** | **3.1** | **0.0** | **3.1** | **3.1** | **0.0** |

In these tables, [$R$] indicates a reversal of the pumping direction to study effects that depend on spin polarization, such as heading errors. To change the rotating field alignment from "$\mathbf{B_m}$ initiates from X-axis" to "$\mathbf{B_m}$ initiates from Y-axis", an additional 90° phase shift is applied to $\phi_x$ and $\phi_y$, respectively.

| $\mathbf{B_m}$ initiates from X-axis | | **Experiment (in units of nT)** | | | | | **Density Matrix (in units of nT)** | | | | |
|---|---|---|---|---|---|---|---|---|---|---|---|
| **Shot No.** | $\beta$ | **Slope** | $B_{Berry}$ | $B_{DH}$ | $B_{SH}$ | $B_{PD}$ | **Slope** | $B_{Berry}$ | $B_{DH}$ | $B_{SH}$ | $B_{PD}$ |
| 1 | 24° | 7.7 | 5.2 | 2.5 | 2.1 | 0.4 | 7.8 | 4.7 | 3.1 | 3.1 | 0.0 |
| 2 | | -2.4 | -5.2 | 2.8 | 2.1 | 0.7 | -1.7 | -4.7 | 3.0 | 3.1 | 0.0 |
| 3 | | 6.9 | 5.2 | 1.7 | 2.1 | -0.4 | 7.8 | 4.7 | 3.1 | 3.1 | 0.0 |
| 4 | | -3.7 | -5.2 | 1.5 | 2.1 | -0.7 | -1.6 | -4.7 | 3.1 | 3.1 | 0.1 |
| **Average** | | **2.1** | **0.0** | **2.1** | **2.1** | **0.0** | **3.1** | **0.0** | **3.1** | **3.1** | **0.0** |
| 1 | 24° Reverse pump | 2.9 | 5.2 | -2.3 | -2.6 | 0.3 | 1.7 | 4.7 | -3.0 | -3.1 | 0.1 |
| 2 | | -8.3 | -5.2 | -3.1 | -2.6 | -0.5 | -7.8 | -4.7 | -3.1 | -3.1 | 0.0 |
| 3 | | 2.4 | 5.2 | -2.8 | -2.6 | -0.3 | 1.6 | 4.7 | -3.1 | -3.1 | 0.0 |
| 4 | | -7.3 | -5.2 | -2.1 | -2.6 | 0.5 | -7.8 | -4.7 | -3.1 | -3.1 | 0.0 |
| **Average** | | **-2.6** | **0.0** | **-2.6** | **-2.6** | **0.0** | **-3.1** | **0.0** | **-3.1** | **-3.1** | **0.0** |
| 1 | 0° | 5.0 | 5.3 | -0.3 | -0.4 | 0.1 | 4.7 | 4.7 | 0.0 | 0.0 | 0.0 |
| 2 | | -5.2 | -5.3 | 0.0 | -0.4 | 0.4 | -4.7 | -4.7 | 0.0 | 0.0 | 0.0 |
| 3 | | 4.8 | 5.3 | -0.5 | -0.4 | -0.1 | 4.7 | 4.7 | 0.0 | 0.0 | 0.0 |
| 4 | | -6.0 | -5.3 | -0.8 | -0.4 | -0.4 | -4.7 | -4.7 | 0.0 | 0.0 | 0.0 |
| **Average** | | **-0.4** | **0.0** | **-0.4** | **-0.4** | **0.0** | **0.0** | **0.0** | **0.0** | **0.0** | **0.0** |
| 1 | 0° Reverse pump | 5.5 | 5.2 | 0.3 | 0.5 | -0.2 | 4.7 | 4.7 | 0.0 | 0.0 | 0.0 |
| 2 | | -5.3 | -5.2 | -0.1 | 0.5 | -0.6 | -4.7 | -4.7 | 0.0 | 0.0 | 0.0 |
| 3 | | 5.8 | 5.2 | 0.6 | 0.5 | 0.1 | 4.7 | 4.7 | 0.0 | 0.0 | 0.0 |
| 4 | | -4.1 | -5.2 | 1.1 | 0.5 | 0.6 | -4.7 | -4.7 | 0.0 | 0.0 | 0.0 |
| **Average** | | **0.5** | **0.0** | **0.5** | **0.5** | **0.0** | **0.0** | **0.0** | **0.0** | **0.0** | **0.0** |
| 1 | -24° | 2.6 | 5.3 | -2.7 | -2.8 | 0.2 | 1.6 | 4.7 | -3.1 | -3.1 | -0.1 |
| 2 | | -7.6 | -5.3 | -2.3 | -2.8 | 0.5 | -7.8 | -4.7 | -3.1 | -3.1 | 0.0 |
| 3 | | 2.3 | 5.3 | -3.0 | -2.8 | -0.2 | 1.7 | 4.7 | -3.0 | -3.1 | 0.0 |
| 4 | | -8.6 | -5.3 | -3.3 | -2.8 | -0.5 | -7.8 | -4.7 | -3.1 | -3.1 | 0.0 |
| **Average** | | **-2.8** | **0.0** | **-2.8** | **-2.8** | **0.0** | **-3.1** | **0.0** | **-3.1** | **-3.1** | **0.0** |
| 1 | -24° Reverse pump | 8.3 | 5.2 | 3.1 | 3.0 | 0.1 | 7.8 | 4.7 | 3.1 | 3.1 | 0.0 |
| 2 | | -2.6 | -5.2 | 2.6 | 3.0 | -0.4 | -1.6 | -4.7 | 3.1 | 3.1 | 0.1 |
| 3 | | 8.1 | 5.2 | 2.9 | 3.0 | -0.1 | 7.8 | 4.7 | 3.1 | 3.1 | 0.0 |
| 4 | | -1.8 | -5.2 | 3.4 | 3.0 | 0.4 | -1.7 | -4.7 | 3.0 | 3.1 | 0.0 |
| **Average** | | **3.0** | **0.0** | **3.0** | **3.0** | **0.0** | **3.1** | **0.0** | **3.1** | **3.1** | **0.0** |

**Supplementary Table 5**: **Transverse magnetic fields** measurement without altering the phases of the rotating field during each panorama measurement. $B_x$ subtract by 8.2 nT, $B_y$ subtract by 12.5 nT. $B_x$ results are similar to the density matrix simulation result, which are close to zero. $B_y$ has a slight magnetic field offset compared with the density matrix simulation result. Here the atoms are pumped along the x-direction and probed along the y-direction. There is a probe beam heading error effect along the y-direction. Based on the probe beam heading error equation, we can estimate the probe beam tilts approximately 1° towards the z-axis.

| | | **$B_m$ initiates from Y-axis** | | | | **$B_m$ initiates from X-axis** | | | |
|---|---|---|---|---|---|---|---|---|---|
| **Shot No.** | $\beta$ | **Experiment** | | **Density Matrix** | | **Experiment** | | **Density Matrix** | |
| | | $B_x$ (nT) | $B_y$ (nT) | $B_x$ (nT) | $B_y$ (nT) | $B_x$ (nT) | $B_y$ (nT) | $B_x$ (nT) | $B_y$ (nT) |
| 1 | 24 ° | -0.2 | -3.3 | 0.1 | -0.2 | -0.5 | -2.7 | 0.1 | 0.2 |
| 2 | | 1.1 | 0.6 | 0.1 | 0.3 | 0.1 | 1.2 | 0.0 | 0.0 |
| 3 | | -0.2 | -2.3 | 0.0 | 0.0 | -0.1 | -3.0 | 0.0 | 0.0 |
| 4 | | 0.0 | 2.1 | 0.0 | 0.0 | 0.6 | 2.0 | 0.1 | 0.2 |
| **Average** | | **0.2** | **-0.7** | **0.0** | **-0.1** | **0.0** | **-0.7** | **0.0** | **0.0** |
| 1 | 24 ° [R] | 0.3 | -0.8 | -0.1 | -0.3 | -0.4 | -1.6 | 0.0 | 0.0 |
| 2 | | 0.3 | 3.1 | -0.1 | 0.2 | -0.2 | 2.6 | 0.0 | -0.2 |
| 3 | | -0.6 | -2.4 | 0.0 | 0.0 | -0.1 | -1.7 | 0.0 | -0.2 |
| 4 | | -0.1 | 2.4 | 0.0 | 0.0 | 0.1 | 2.9 | -0.1 | 0.0 |
| **Average** | | **0.0** | **0.6** | **0.0** | **-0.1** | **-0.1** | **0.6** | **0.0** | **0.0** |
| 1 | 0 ° | -0.1 | -1.5 | 0.1 | -0.1 | -0.1 | -0.8 | 0.0 | 0.0 |
| 2 | | 0.5 | 0.4 | 0.1 | 0.1 | 0.1 | 0.8 | 0.0 | 0.0 |
| 3 | | 0.1 | -0.3 | -0.1 | -0.1 | 0.1 | -1.0 | 0.0 | 0.0 |
| 4 | | -0.2 | 1.6 | -0.1 | 0.0 | 0.2 | 1.3 | 0.0 | 0.0 |
| **Average** | | **0.1** | **0.0** | **0.1** | **0.0** | **0.1** | **0.1** | **0.0** | **0.0** |
| 1 | 0 ° [R] | 0.3 | -0.5 | -0.1 | -0.1 | -0.3 | -0.9 | 0.0 | 0.0 |
| 2 | | -0.1 | 1.4 | -0.1 | 0.0 | -0.1 | 1.0 | 0.0 | 0.0 |
| 3 | | -0.5 | -1.7 | 0.1 | -0.1 | -0.3 | -1.2 | 0.0 | 0.0 |
| 4 | | 0.0 | 0.3 | 0.1 | 0.1 | 0.0 | 1.1 | 0.0 | 0.0 |
| **Average** | | **-0.1** | **-0.1** | **-0.1** | **0.0** | **-0.2** | **0.0** | **0.0** | **0.0** |
| 1 | -24 ° | 0.4 | 0.5 | 0.0 | 0.0 | 0.1 | 1.0 | 0.0 | -0.2 |
| 2 | | 0.1 | 0.1 | 0.0 | 0.0 | 0.0 | 0.2 | -0.1 | 0.0 |
| 3 | | 0.2 | 1.8 | -0.1 | -0.3 | 0.3 | 0.8 | 0.0 | 0.0 |
| 4 | | -0.6 | 1.1 | -0.1 | 0.2 | -0.2 | 0.8 | 0.0 | -0.2 |
| **Average** | | **0.0** | **0.9** | **0.0** | **0.1** | **0.1** | **0.7** | **0.0** | **0.0** |
| 1 | -24 ° [R] | 0.0 | 0.2 | 0.0 | 0.0 | -0.7 | -0.1 | 0.0 | 0.0 |
| 2 | | -0.1 | -0.2 | 0.0 | 0.0 | -0.1 | -0.8 | 0.1 | 0.2 |
| 3 | | -0.6 | -1.2 | 0.1 | -0.2 | -0.2 | -0.4 | 0.1 | 0.2 |
| 4 | | 0.2 | -1.7 | 0.1 | 0.3 | 0.0 | -0.8 | 0.0 | 0.0 |
| **Average** | | **-0.1** | **-0.7** | **0.0** | **0.1** | **-0.2** | **-0.5** | **0.0** | **0.0** |

**Supplementary Table 6**: **Total magnetic field** measurement with the four-shot scheme. All the slopes are subtracted by 0.5 nT to remove $\delta b_z$. This table is similar to Table 4. However, the data in this table are measured using the four-shot scheme. The total magnetic field measurement is not significantly affected by the residual eddy current magnetic field.

| $\mathbf{B_m}$ initiates from Y-axis | | **Experiment (in units of nT)** | | | | | **Density Matrix (in units of nT)** | | | | |
|---|---|---|---|---|---|---|---|---|---|---|---|
| **Shot No.** | $\beta$ | **Slope** | $B_{Berry}$ | $B_{DH}$ | $B_{SH}$ | $B_{PD}$ | **Slope** | $B_{Berry}$ | $B_{DH}$ | $B_{SH}$ | $B_{PD}$ |
| 1 | | 9.5 | 5.1 | 4.4 | 2.1 | 2.3 | 10.5 | 4.8 | 5.8 | 3.1 | 2.7 |
| 2 | | -0.7 | -5.1 | 4.4 | 2.1 | 2.4 | 1.0 | -4.8 | 5.8 | 3.1 | 2.7 |
| 3 | 24° | 4.9 | 5.1 | -0.2 | 2.1 | -2.3 | 5.1 | 4.8 | 0.4 | 3.1 | -2.7 |
| 4 | | -5.4 | -5.1 | -0.3 | 2.1 | -2.4 | -4.4 | -4.8 | 0.4 | 3.1 | -2.7 |
| **Average** | | **2.1** | **0.0** | **2.1** | **2.1** | **0.0** | **3.1** | **0.0** | **3.1** | **3.1** | **0.0** |
| 1 | | -0.1 | 4.9 | -5.0 | -2.5 | -2.5 | -1.0 | 4.8 | -5.8 | -3.1 | -2.7 |
| 2 | | -9.5 | -4.9 | -4.7 | -2.5 | -2.2 | -10.5 | -4.8 | -5.8 | -3.1 | -2.7 |
| 3 | 24° Reverse pump | 4.9 | 4.9 | 0.1 | -2.5 | 2.5 | 4.4 | 4.8 | -0.4 | -3.1 | 2.7 |
| 4 | | -5.1 | -4.9 | -0.3 | -2.5 | 2.2 | -5.1 | -4.8 | -0.4 | -3.1 | 2.7 |
| **Average** | | **-2.5** | **0.0** | **-2.5** | **-2.5** | **0.0** | **-3.1** | **0.0** | **-3.1** | **-3.1** | **0.0** |
| 1 | | 7.5 | 5.2 | 2.3 | -0.3 | 2.6 | 7.7 | 4.8 | 3.0 | 0.0 | 3.0 |
| 2 | | -2.9 | -5.2 | 2.3 | -0.3 | 2.6 | -1.8 | -4.8 | 3.0 | 0.0 | 3.0 |
| 3 | 0° | 2.3 | 5.2 | -2.9 | -0.3 | -2.6 | 1.8 | 4.8 | -3.0 | 0.0 | -3.0 |
| 4 | | -8.1 | -5.2 | -2.9 | -0.3 | -2.6 | -7.7 | -4.8 | -3.0 | 0.0 | -3.0 |
| **Average** | | **-0.3** | **0.0** | **-0.3** | **-0.3** | **0.0** | **0.0** | **0.0** | **0.0** | **0.0** | **0.0** |
| 1 | | 2.7 | 5.2 | -2.5 | 0.1 | -2.7 | 1.8 | 4.8 | -3.0 | 0.0 | -3.0 |
| 2 | | -7.6 | -5.2 | -2.4 | 0.1 | -2.5 | -7.7 | -4.8 | -3.0 | 0.0 | -3.0 |
| 3 | 0° Reverse pump | 8.0 | 5.2 | 2.8 | 0.1 | 2.7 | 7.7 | 4.8 | 3.0 | 0.0 | 3.0 |
| 4 | | -2.6 | -5.2 | 2.6 | 0.1 | 2.5 | -1.8 | -4.8 | 3.0 | 0.0 | 3.0 |
| **Average** | | **0.1** | **0.0** | **0.1** | **0.1** | **0.0** | **0.0** | **0.0** | **0.0** | **0.0** | **0.0** |
| 1 | | 4.8 | 5.3 | -0.5 | -2.8 | 2.3 | 4.4 | 4.8 | -0.4 | -3.1 | 2.7 |
| 2 | | -5.9 | -5.3 | -0.6 | -2.8 | 2.2 | -5.1 | -4.8 | -0.4 | -3.1 | 2.7 |
| 3 | -24° | 0.3 | 5.3 | -5.0 | -2.8 | -2.3 | -1.0 | 4.8 | -5.8 | -3.1 | -2.7 |
| 4 | | -10.2 | -5.3 | -4.9 | -2.8 | -2.2 | -10.5 | -4.8 | -5.8 | -3.1 | -2.7 |
| **Average** | | **-2.8** | **0.0** | **-2.8** | **-2.8** | **0.0** | **-3.1** | **0.0** | **-3.1** | **-3.1** | **0.0** |
| 1 | | 6.0 | 5.0 | 1.0 | 3.2 | -2.3 | 5.1 | 4.8 | 0.4 | 3.1 | -2.7 |
| 2 | | -4.1 | -5.0 | 0.9 | 3.2 | -2.3 | -4.4 | -4.8 | 0.4 | 3.1 | -2.7 |
| 3 | -24° Reverse pump | 10.5 | 5.0 | 5.5 | 3.2 | 2.3 | 10.5 | 4.8 | 5.8 | 3.1 | 2.7 |
| 4 | | 0.5 | -5.0 | 5.5 | 3.2 | 2.3 | 1.0 | -4.8 | 5.8 | 3.1 | 2.7 |
| **Average** | | **3.2** | **0.0** | **3.2** | **3.2** | **0.0** | **3.1** | **0.0** | **3.1** | **3.1** | **0.0** |

| $\mathbf{B_m}$ initiates from X-axis | | **Experiment (in units of nT)** | | | | | **Density Matrix (in units of nT)** | | | | |
|---|---|---|---|---|---|---|---|---|---|---|---|
| **Shot No.** | $\beta$ | **Slope** | $B_{Berry}$ | $B_{DH}$ | $B_{SH}$ | $B_{PD}$ | **Slope** | $B_{Berry}$ | $B_{DH}$ | $B_{SH}$ | $B_{PD}$ |
| 1 | | 7.3 | 5.4 | 1.9 | 1.7 | 0.1 | 7.8 | 4.7 | 3.1 | 3.1 | 0.0 |
| 2 | | -3.3 | -5.4 | 2.1 | 1.7 | 0.4 | -1.7 | -4.7 | 3.0 | 3.1 | 0.0 |
| 3 | 24° | 7.0 | 5.4 | 1.6 | 1.7 | -0.2 | 7.8 | 4.7 | 3.1 | 3.1 | 0.0 |
| 4 | | -4.1 | -5.4 | 1.3 | 1.7 | -0.4 | -1.6 | -4.7 | 3.1 | 3.1 | 0.1 |
| **Average** | | **1.7** | **0.0** | **1.7** | **1.7** | **0.0** | **3.1** | **0.0** | **3.1** | **3.1** | **0.0** |
| 1 | | 2.9 | 5.3 | -2.4 | -2.4 | 0.0 | 1.7 | 4.7 | -3.0 | -3.1 | 0.1 |
| 2 | 24° | -8.0 | -5.3 | -2.8 | -2.4 | -0.4 | -7.8 | -4.7 | -3.1 | -3.1 | 0.0 |
| 3 | Reverse pump | 2.9 | 5.3 | -2.4 | -2.4 | 0.0 | 1.6 | 4.7 | -3.1 | -3.1 | 0.0 |
| 4 | | -7.2 | -5.3 | -2.0 | -2.4 | 0.4 | -7.8 | -4.7 | -3.1 | -3.1 | 0.0 |
| **Average** | | **-2.4** | **0.0** | **-2.4** | **-2.4** | **0.0** | **-3.1** | **0.0** | **-3.1** | **-3.1** | **0.0** |
| 1 | | 5.3 | 5.2 | 0.2 | 0.0 | 0.1 | 4.7 | 4.7 | 0.0 | 0.0 | 0.0 |
| 2 | | -4.7 | -5.2 | 0.4 | 0.0 | 0.4 | -4.7 | -4.7 | 0.0 | 0.0 | 0.0 |
| 3 | 0° | 5.1 | 5.2 | 0.0 | 0.0 | -0.1 | 4.7 | 4.7 | 0.0 | 0.0 | 0.0 |
| 4 | | -5.5 | -5.2 | -0.4 | 0.0 | -0.4 | -4.7 | -4.7 | 0.0 | 0.0 | 0.0 |
| **Average** | | **0.0** | **0.0** | **0.0** | **0.0** | **0.0** | **0.0** | **0.0** | **0.0** | **0.0** | **0.0** |
| 1 | | 6.1 | 5.5 | 0.6 | 0.6 | 0.0 | 4.7 | 4.7 | 0.0 | 0.0 | 0.0 |
| 2 | 0° | -5.4 | -5.5 | 0.1 | 0.6 | -0.5 | -4.7 | -4.7 | 0.0 | 0.0 | 0.0 |
| 3 | Reverse pump | 6.1 | 5.5 | 0.6 | 0.6 | 0.0 | 4.7 | 4.7 | 0.0 | 0.0 | 0.0 |
| 4 | | -4.5 | -5.5 | 1.0 | 0.6 | 0.5 | -4.7 | -4.7 | 0.0 | 0.0 | 0.0 |
| **Average** | | **0.6** | **0.0** | **0.6** | **0.6** | **0.0** | **0.0** | **0.0** | **0.0** | **0.0** | **0.0** |
| 1 | | 2.5 | 5.3 | -2.8 | -2.9 | 0.1 | 1.6 | 4.7 | -3.1 | -3.1 | -0.1 |
| 2 | | -7.8 | -5.3 | -2.5 | -2.9 | 0.4 | -7.8 | -4.7 | -3.1 | -3.1 | 0.0 |
| 3 | -24° | 2.3 | 5.3 | -3.0 | -2.9 | -0.1 | 1.7 | 4.7 | -3.0 | -3.1 | 0.0 |
| 4 | | -8.6 | -5.3 | -3.3 | -2.9 | -0.4 | -7.8 | -4.7 | -3.1 | -3.1 | 0.0 |
| **Average** | | **-2.9** | **0.0** | **-2.9** | **-2.9** | **0.0** | **-3.1** | **0.0** | **-3.1** | **-3.1** | **0.0** |
| 1 | | 8.0 | 5.1 | 2.9 | 2.9 | 0.0 | 7.8 | 4.7 | 3.1 | 3.1 | 0.0 |
| 2 | -24° | -2.6 | -5.1 | 2.5 | 2.9 | -0.5 | -1.6 | -4.7 | 3.1 | 3.1 | 0.1 |
| 3 | Reverse pump | 8.0 | 5.1 | 2.9 | 2.9 | 0.0 | 7.8 | 4.7 | 3.1 | 3.1 | 0.0 |
| 4 | | -1.7 | -5.1 | 3.4 | 2.9 | 0.5 | -1.7 | -4.7 | 3.0 | 3.1 | 0.0 |
| **Average** | | **2.9** | **0.0** | **2.9** | **2.9** | **0.0** | **3.1** | **0.0** | **3.1** | **3.1** | **0.0** |

**Supplementary Table 7**: **Transverse magnetic fields** measurement with the four-shot scheme. $B_x$ subtract by 7.6 nT, $B_y$ subtract by 12 nT. This table is similar to Supplementary Table 5. The difference is the data in this table are measured with the four-shot scheme, and there is a residual eddy current magnetic field due to the imperfection of the "cosine switch". The eddy current systematic can be canceled out by averaging through four shots.

| | | **$B_m$ initiates from Y-axis** | | | | **$B_m$ initiates from X-axis** | | | |
|---|---|---|---|---|---|---|---|---|---|
| **Shot No.** | $\beta$ | **Experiment** | | **Density Matrix** | | **Experiment** | | **Density Matrix** | |
| | | $B_x$ (nT) | $B_y$ (nT) | $B_x$ (nT) | $B_y$ (nT) | $B_x$ (nT) | $B_y$ (nT) | $B_x$ (nT) | $B_y$ (nT) |
| **1** | 24 ° | -16.6 | -12.2 | 0.1 | -0.2 | 15.9 | -11.2 | 0.1 | 0.2 |
| 2 | | -6.2 | 16.1 | 0.1 | 0.3 | 7.7 | 15.5 | 0.0 | 0.0 |
| 3 | | 16.0 | 5.2 | 0.0 | 0.0 | -15.6 | 5.0 | 0.0 | 0.0 |
| 4 | | 6.7 | -12.4 | 0.0 | 0.0 | -7.7 | -12.2 | 0.1 | 0.2 |
| **Average** | | **0.0** | **-0.8** | **0.0** | **-0.1** | **0.1** | **-0.7** | **0.0** | **0.0** |
| 1 | 24 ° [R] | -13.7 | -8.9 | -0.1 | -0.3 | 15.7 | -9.7 | 0.0 | 0.0 |
| 2 | | -9.2 | 19.5 | -0.1 | 0.2 | 7.5 | 17.7 | 0.0 | -0.2 |
| 3 | | 14.6 | 5.0 | 0.0 | 0.0 | -15.8 | 5.7 | 0.0 | -0.2 |
| 4 | | 8.1 | -12.7 | 0.0 | 0.0 | -8.6 | -11.9 | -0.1 | 0.0 |
| **Average** | | **0.0** | **0.7** | **0.0** | **-0.1** | **-0.3** | **0.5** | **0.0** | **0.0** |
| 1 | 0 ° | -14.7 | -10.0 | 0.1 | -0.1 | 15.6 | -8.7 | 0.0 | 0.0 |
| 2 | | -7.8 | 16.6 | 0.1 | 0.1 | 8.8 | 16.5 | 0.0 | 0.0 |
| 3 | | 15.7 | 7.0 | -0.1 | -0.1 | -14.9 | 5.9 | 0.0 | 0.0 |
| 4 | | 7.5 | -13.1 | -0.1 | 0.0 | -8.9 | -14.0 | 0.0 | 0.0 |
| **Average** | | **0.2** | **0.1** | **0.1** | **0.0** | **0.1** | **-0.1** | **0.0** | **0.0** |
| 1 | 0 ° [R] | -14.5 | -8.9 | -0.1 | -0.1 | 16.2 | -9.7 | 0.0 | 0.0 |
| 2 | | -8.3 | 17.3 | -0.1 | 0.0 | 7.6 | 15.5 | 0.0 | 0.0 |
| 3 | | 15.4 | 5.9 | 0.1 | -0.1 | -15.7 | 7.0 | 0.0 | 0.0 |
| 4 | | 7.6 | -14.4 | 0.1 | 0.1 | -8.1 | -13.1 | 0.0 | 0.0 |
| **Average** | | **0.0** | **0.0** | **-0.1** | **0.0** | **0.0** | **-0.1** | **0.0** | **0.0** |
| 1 | -24 ° | -14.1 | -8.8 | 0.0 | 0.0 | 16.9 | -8.0 | 0.0 | -0.2 |
| 2 | | -9.2 | 15.1 | 0.0 | 0.0 | 6.9 | 14.6 | -0.1 | 0.0 |
| 3 | | 15.0 | 9.5 | -0.1 | -0.3 | -15.6 | 8.8 | 0.0 | 0.0 |
| 4 | | 8.1 | -13.4 | -0.1 | 0.2 | -8.0 | -13.3 | 0.0 | -0.2 |
| **Average** | | **0.0** | **0.6** | **0.0** | **0.1** | **0.0** | **0.5** | **0.0** | **0.0** |
| 1 | -24 ° [R] | -14.6 | -8.1 | 0.0 | 0.0 | 16.0 | -8.3 | 0.0 | 0.0 |
| 2 | | -8.4 | 15.9 | 0.0 | 0.0 | 7.6 | 14.5 | 0.1 | 0.2 |
| 3 | | 14.8 | 5.6 | 0.1 | -0.2 | -15.6 | 6.5 | 0.1 | 0.2 |
| 4 | | 8.2 | -17.7 | 0.1 | 0.3 | -8.4 | -15.9 | 0.0 | 0.0 |
| **Average** | | **0.0** | **-1.1** | **0.0** | **0.1** | **-0.1** | **-0.8** | **0.0** | **0.0** |

## Supplementary References


[1] Auzinsh, M., Budker, D. & Rochester, S. *Optically polarized atoms: understanding light-atom interactions* (Oxford University Press, 2010).

[2] Savukov, I. M., Seltzer, S., Romalis, M. & Sauer, K. Tunable atomic magnetometer for detection of radio-frequency magnetic fields. *Physical Review Letters* **95**, 063004 (2005).

[3] Kay, S. M. *Fundamentals of statistical signal processing* (Prentice Hall PTR, 1993).

[4] Seltzer, S. J. *Developments in alkali-metal atomic magnetometry* (Princeton University, 2008).

[5] Lee, W. *et al.* Heading errors in all-optical alkali-metal-vapor magnetometers in geomagnetic fields. *Physical Review A* **103**, 063103 (2021).

[6] Bize, S. *et al.* High-accuracy measurement of the 87rb ground-state hyperfine splitting in an atomic fountain. *EPL (Europhysics Letters)* **45**, 558 (1999).

[7] Alldredge, L. A proposed automatic standard magnetic observatory. *Journal of Geophysical Research* **65**, 3777–3786 (1960).

[8] Appelt, S. *et al.* Theory of spin-exchange optical pumping of 3 he and 129 xe. *Physical Review A* **58**, 1412 (1998).

[9] Walker, T. G. & Happer, W. Spin-exchange optical pumping of noble-gas nuclei. *Reviews of Modern Physics* **69**, 629 (1997).

[10] Seltzer, S. & Romalis, M. Unshielded three-axis vector operation of a spin-exchange-relaxation-free atomic magnetometer. *Applied Physics Letters* **85**, 4804–4806 (2004).

[11] Vershovskii, A. *et al.* Fast three-component magnetometer-variometer based on a cesium sensor. *Technical Physics* **51**, 112–117 (2006).

[12] Patton, B., Zhivun, E., Hovde, D. & Budker, D. All-optical vector atomic magnetometer. *Physical Review Letters* **113**, 013001 (2014).

[13] Afach, S. *et al.* Highly stable atomic vector magnetometer based on free spin precession. *Optics Express* **23**, 22108–22115 (2015).

[14] Zhao, Q., Fan, B., Wang, S. & Wang, L. A vector atomic magnetometer based on the spin self-sustaining larmor method. *Journal of Magnetism and Magnetic Materials* **481**, 257–261 (2019).

[15] Pyragius, T., Florez, H. M. & Fernholz, T. Voigt-effect-based three-dimensional vector magnetometer. *Physical Review A* **100**, 023416 (2019).

[16] Meng, X. *et al.* Machine learning assisted vector atomic magnetometry. *Nature Communications* **14**, 6105 (2023).
